# First person – Ana Filipa Castro

**DOI:** 10.1242/dmm.052960

**Published:** 2026-04-30

## Abstract

First Person is a series of interviews with the first authors of a selection of papers published in Disease Models & Mechanisms, helping researchers promote themselves alongside their papers. Ana Filipa Castro is first author on ‘ Embryonic spinocerebellar ataxia type 37-associated AUUUC repeat RNA causes neurodevelopmental defects’, published in DMM. Ana Filipa is a PhD student in the lab of Isabel Silveira at the Institute for Molecular and Cell Biology (IBMC) and the i3S-Institute for Research and Innovation in Health Sciences, University of Porto, Porto, Portugal, investigating the cellular and molecular mechanisms that underlie neurodegenerative diseases caused by repeat mutations, with a focus on generating animal models and developing therapeutic strategies.

**Figure:**
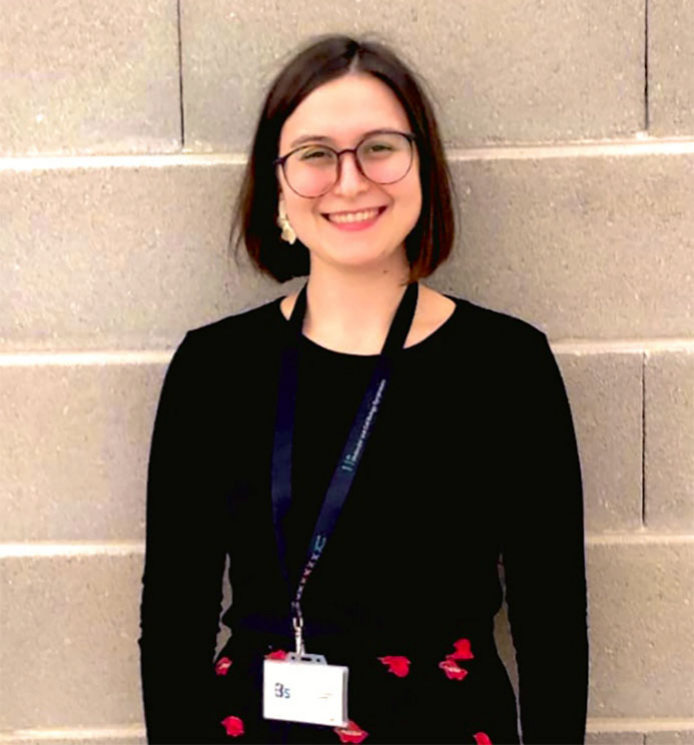
Ana Filipa Castro

**Who or what inspired you to become a scientist?**

To be honest, it wasn't a single person or specific moment that inspired me to become a scientist. My interest developed gradually, especially during my biology classes in high school. I became really fascinated by cellular and molecular processes that happen beyond what we can see. Understanding how these invisible mechanisms explain how the human body works really captured my curiosity and motivated me to pursue science further.

**What is the main question or challenge in disease biology you are addressing in this paper? How did you go about investigating your question or challenge?**

Our main question was to understand how a repeat mutation in a non-coding DNA region leads to spinocerebellar ataxia type 37 (SCA37) , a neurodegenerative disease characterized by progressive loss of cerebellar Purkinje neurons and motor incoordination later in life. What makes this particularly intriguing is that the gene carrying this repeat mutation is more highly expressed during neurodevelopment than in adulthood. This led us to hypothesize that the mutant RNA repeat may cause neuronal defects much earlier in life, even though symptoms appear decades later. To address this, we microinjected the mutant RNA repeat into zebrafish embryos and found that its early developmental expression is sufficient to induce axonal defects, likely contributing to the locomotor dysfunction observed later in adulthood in both the model and patients.

**How would you explain the main findings of your paper to non-scientific family and friends?**

The disease we study is an inherited condition caused by a genetic alteration that gradually damages neurons. Over time, people with this disease lose control of their movements, making simple tasks like walking, speaking or eating very difficult. Because of this, it is very important to understand how this damage is caused and how to stop it. In our research, we discovered that this genetic alteration disrupts an important process in neuronal cells. We also found something hopeful; that is, damage may begin much earlier in life than symptoms first appear. This means there could be a long time window, during which treatment might help to slow down the disease or delay its effects before it becomes severe.

Our results indicate that there is a long interval between the start of neuronal defects and the appearance of clinical symptoms, which opens avenues for timely treatment to prevent disease progression and worsening

**What are the potential implications of these results for disease biology and the possible impact on patients?**

One major problem of neurodegenerative diseases is that, when patients present clinical symptoms, a substantial number of neurons have already been lost and cannot be replaced. Importantly, our results indicate that there is a long interval between the start of neuronal defects and the appearance of clinical symptoms, which opens avenues for timely treatment to prevent disease progression and worsening. As this is a hereditary disease, mutation carriers can be identified before the onset of symptoms, so they can benefit from early therapeutic intervention, preventing more-severe disease outcomes. In our work, we have also generated a zebrafish model that can be used for high-throughput screening of potential therapeutic targets. Moreover, our results may be extended to the other seven neurological diseases caused by similar repeat mutations.

DMM is a prestigious open-access journal with high-quality publications […] allowing it to reach a diverse scientific audience

**Figure:**
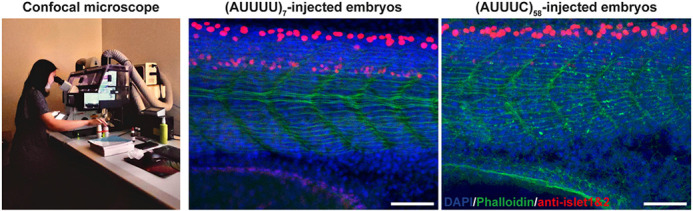
**Initial observation of motor neuron somas and muscle fibres in our zebrafish model by using an SP8 confocal microscope.** Whole-mount immunofluorescence of zebrafish embryos 24 h post fertilization, which had been microinjected with the non-pathogenic (AUUUU)_7_ (middle image) or the pathogenic (AUUUC)_58_ (right image) RNA. Antibodies against Islet 1 and Islet 2 were used to stain for primary motoneurons (red) to investigate neuronal and muscle alterations; F-actin was stained with phalloidin (green), nuclei were stained with DAPI (blue). As no clear alterations were observed in motor neuron cell bodies or muscle fibers, axon length of primary motor neurons was analyzed. Scale bars: 50 µm.

**Why did you choose DMM for your paper?**

The scope of DMM is fully aligned with our work, which focused on generating a zebrafish model to investigate the mechanisms underlying a human neurodegenerative disease. Moreover, DMM is a prestigious open-access journal with high-quality publications. A particular advantage of this journal is its wide range of topics, allowing it to reach a diverse scientific audience. Science is made to be broadly shared, and DMM provides an ideal platform to disseminate our findings.

**Given your current role, what challenges do you face and what changes could improve the professional lives of other scientists in this role?**

Currently, I am finishing my PhD focused on a rare neurodegenerative disease. Many researchers like me face several challenges in investigating the mechanisms underlying neurodegenerative diseases. Access to human brain tissue is extremely limited and, when available, it represents only the final stage of disease, making it difficult to address these questions. More animal models and new technologies, like reprogramming patient-derived cells into specific neurons and reconstructing more-realistic neuronal networks, would advance research in the field but, for that, increased funding and resources would be required. Personally, I think a researcher faces multiple challenges simultaneously, including planning projects, performing experiments, writing manuscripts, applying for funding and trying to maintain a ‘normal’ life. Therefore, I believe that it is important to create conditions to retain people in academia.

**What's next for you?**

I am confident in pursuing an academic career as a scientist in the field of repeat mutation-associated neurodegenerative diseases. I am particularly interested in animal modelling and the development of therapeutic strategies. With this focus, I am currently seeking postdoc positions that will allow me to evolve in these areas.

**Tell us something interesting about yourself that wouldn't be on your CV**

Outside of the lab, I'm a big fan of live music. Whenever possible, I don't hesitate to pause my experiments to catch a show and then return to the lab as needed. Listening to music energizes me and keeps me focused, helping me to clear my mind and dive into work.
